# Reduced‐Frequency GLP1 Therapy Maintains Weight, Body Composition, and Metabolic Syndrome Improvements: A Case Series

**DOI:** 10.1002/oby.70137

**Published:** 2026-02-24

**Authors:** Michelle Wong, Ash Wu, Pawanjot K. Garhe, Mitch Biermann

**Affiliations:** ^1^ Department of Internal Medicine Scripps Clinic San Diego California USA; ^2^ Samuel Merritt University Oakland California USA; ^3^ University of California San Diego California USA; ^4^ Scripps Whittier Diabetes Institute San Diego California USA

**Keywords:** GLP1 maintenance dosing, GLP1 tapering protocol, reduced‐frequency GLP1 therapy, semaglutide, sustainable obesity treatment, tirzepatide, weight loss

## Abstract

**Objective:**

This study aimed to evaluate whether reduced‐frequency dosing of GLP1 receptor agonists maintains weight loss, body composition, and metabolic syndrome improvements following successful initial treatment with standard weekly therapy.

**Methods:**

This retrospective case series included 30 adults who achieved weight plateau while on weekly semaglutide or tirzepatide. Patients transitioned to reduced‐frequency dosing (usually every other week) at their existing dose. Data were collected at three time points: pre‐treatment, plateau (weekly dosing), and maintenance (reduced‐frequency dosing). Primary outcome was change in body weight from plateau to maintenance. Secondary outcomes included body composition and metabolic syndrome comorbidities.

**Results:**

Patients maintained reduced‐frequency dosing for an average of 36.3 weeks. Weight decreased from 87.9 ± 2.4 kg at pre‐treatment to 74.1 ± 2.4 kg at plateau and further to 72.4 ± 2.2 kg on maintenance dosing (*p* < 0.01). Total body and truncal fat declined, while skeletal muscle mass stabilized on the reduced‐frequency regimen. Metabolic parameters improved during weekly dosing and these gains were maintained during reduced‐frequency therapy.

**Conclusions:**

In patients with prior weight loss and metabolic improvement on GLP1 therapy, reduced‐frequency maintenance dosing preserved outcomes. These findings support structured de‐escalation as a promising strategy to reduce treatment burden without sacrificing efficacy.

## Study Importance

- What is already known?
  ○ GLP1 receptor agonists induce significant weight loss and improve metabolic health when taken weekly.
  ○ Abrupt discontinuation of GLP1 therapy often leads to weight regain and reversal of benefits.
- What does this study add?
  ○ Patients who transitioned to reduced‐frequency GLP1 dosing maintained weight, body composition, and metabolic markers.
  ○ Structured de‐escalation is feasible in selected patients without significant risk of relapse.
- How might these results change the direction of research or the focus of clinical practice?
  ○ These results support development of evidence‐based tapering protocols for GLP1 therapy.
  ○ Our study may inform cost reduction, improve adherence, and expand access through individualized maintenance regimens.

## 1 Introduction

Incretin mimetics—particularly the glucagon‐like peptide‐1 (GLP1) receptor agonist semaglutide [1] and the dual glucose‐dependent insulinotropic polypeptide (GIP)/GLP1 agonist tirzepatide [2]—offer a noninvasive approach to achieving weight loss approaching that of bariatric surgery, with durable results when administered weekly at the highest tolerated doses. GLP1 therapies also improve several obesity‐related conditions, including metabolic syndrome [3], major adverse cardiac events [4], diabetic kidney disease [5], sleep apnea [6], and metabolic‐associated fatty liver disease [7].

Despite these benefits, uptake remains low. While an estimated 51% of US adults meet eligibility criteria, only 6% report using GLP1 therapies, with notable disparities by sex, race/ethnicity, and socioeconomic status [8, 9]. Barriers include fear of side effects, cost, lack of access to insurance or health care providers, supply limitations, and skepticism regarding long‐term use [10]. These challenges are compounded by the expectation of lifelong therapy, leading many patients to delay initiation and payers to restrict coverage.

Many patients are reluctant to remain on lifelong medications, and in our clinical experience, some report self‐directed rationing or experimentation with alternative dosing regimens. However, no evidence‐based protocols currently guide GLP1 de‐escalation to prevent weight regain or reversal of metabolic improvements. Existing studies focus on abrupt cessation after a fixed treatment duration, consistently demonstrating weight regain and metabolic deterioration [1, 11, 12, 13]. These trials do not assess outcomes in patients selected for de‐escalation based on clinical improvement in body mass index (BMI) or comorbidities.

We hypothesized that reduced‐frequency or lower‐dose GLP1 regimens may be effective in the weight maintenance phase. This is supported by pharmacokinetic models suggesting extended dosing intervals maintain therapeutic efficacy [14] and by data from nonpharmacologic interventions known to modestly stimulate endogenous incretin production. These include pre‐prandial chewing [15], strategic meal sequencing [16], increased intake of protein [17], vegetables [18], fermented or probiotic foods [19, 20], unprocessed foods [21], regular physical activity [22], and improved sleep quality [23, 24].

In this retrospective case series, we examine outcomes in patients who, after achieving weight plateau on GLP1 therapy, transitioned to reduced‐frequency maintenance dosing—typically at nonmaximal doses. We find that these regimens generally maintain weight, body composition, and metabolic outcomes.

## 2 Methods

This retrospective case series was approved by the Scripps Research Institutional Review Board, including the research protocol, statistical analysis plan, and waiver of informed consent.

### 2.1 Patient Selection and Clinical Protocol

Patients were adults in a community/academic primary care obesity practice prescribed GLP1 therapy (semaglutide or tirzepatide) according to FDA‐labeled indications: the presence of type 2 diabetes, BMI ≥ 30 kg/m^2^, or BMI ≥ 27 kg/m^2^ with at least one weight‐related comorbidity. As no standard of care exists for tapering GLP1 therapy, de‐escalation was offered as part of routine clinical care. Patients who reported weight loss plateau (defined as less than 5% variation over a 3‐month period) were invited to switch from standard once‐weekly dosing to reduced‐frequency dosing (every other week) at their current effective dose.

### 2.2 Study Time Points and Measurements

Clinical data were collected at three key time points: Pre‐GLP1 (“Pre”), before initiation of GLP1 therapy; Plateau (“Plat”), at the point of maximum observed weight loss on weekly dosing prior to de‐escalation; and Maintenance (“Maint”), at the most recent follow‐up on reduced‐frequency therapy. At each visit (typically every 3 months), patients were evaluated for body weight (kg) and BMI (kg/m^2^), blood pressure (mm Hg), body composition by bioelectrical impedance (using an InBody 570 analyzer), and metabolic laboratory markers—hemoglobin A1c (HbA1c, %), triglycerides (mg/dL), and high‐density lipoprotein (HDL, mg/dL). GLP1 dosing (dose and frequency) was recorded at each visit. While every‐other‐week dosing was the recommended reduced‐frequency regimen, final dosing frequency was guided by patient preference. Patients who declined reduced‐frequency dosing or who reverted to weekly dosing due to weight regain were recorded.

### 2.3 Statistical Analysis

The prespecified primary outcome and comparison were the within‐subject change in absolute body weight between the plateau and maintenance time points among patients continuing on reduced‐frequency dosing. Weight data were also presented as BMI and percent change in body weight from pre‐GLP1. Secondary comparisons were made between pre‐GLP1 and plateau time points. Paired *t*‐tests were used for all within‐subject comparisons, with statistical significance defined as *p* < 0.05. All continuous data are presented as mean ± standard error of the mean (SEM). Secondary outcomes included body composition analysis (percent body fat, total and truncal fat mass, skeletal muscle mass) and metabolic syndrome markers (HbA1c, systolic and diastolic blood pressure, triglycerides, HDL cholesterol). Additionally, the prevalence of each metabolic syndrome comorbidity was calculated at each time point using standard diagnostic thresholds.

## 3 Results

### 3.1 Patient Characteristics

**FIGURE 1:**
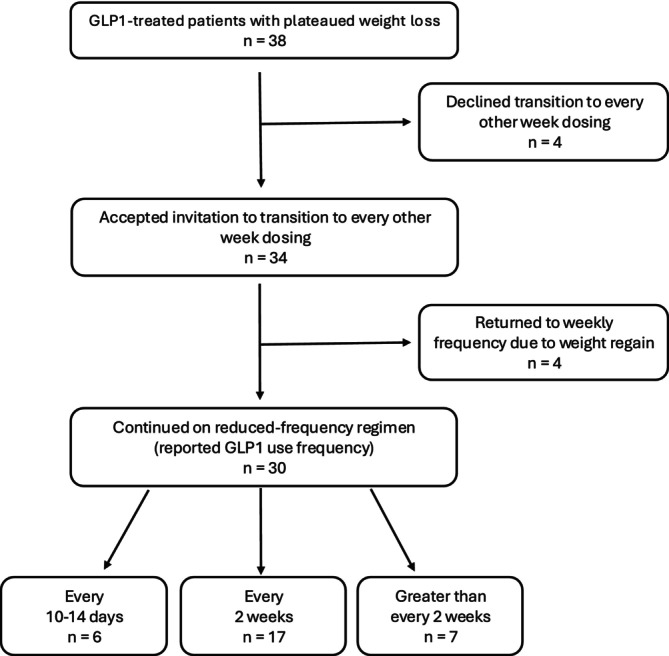
Case flowchart of individuals included and excluded from the study and reduced‐frequency regimen patient‐reported frequencies.

Among the initial 38 participants, 4 participants noted intention to begin reduced‐frequency dosing but reported later they never did so and stayed on weekly dosing, and 4 participants returned to weekly dosing due to weight regain before the first follow‐up visit (Figure 1). Thus 30/34 or 88% of patients did not leave reduced‐frequency dosing due to weight regain and remained on the maintenance dosing regimen, and they were included in the analysis.

The average length of time on the maintenance regimen was 36 weeks with a range of 11–115 weeks. Of the 30 participants studied, 21 were on tirzepatide and 9 were on semaglutide (Table 1). The average dose was 10 mg for tirzepatide and 1.7 mg for semaglutide (rounded to nearest standard dose). Twenty‐four patients reported frequency of injections that was a minimum of 2 weeks apart with the longest interval reported as 6 weeks apart. Six participants reported frequency between 10 and 14 days. The average patient age was 58, average BMI was 30, and 17 patients were male and 13 female. Patients were primarily White (26) and Asian (4).

**TABLE 1:** Patient case data. [Color table can be viewed at wileyonlinelibrary.com]

Case	Age	Sex	GLP1 dose	Frequency	Weeks maint	Pre Wt	Plat Wt	Maint Wt	Pre BMI	Plat BMI	Maint BMI
1	56	F	Tirz. 5	10 days	115	75	65	66	29	25	26
2	56	F	Tirz. 5	2 weeks	55	75	56	56	30	23	23
3	39	M	Sema. 1.7	2 weeks	43	89	75	78	27	23	24
4	70	M	Tirz. 15	2 weeks	54	80	72	70	27	24	24
5	59	M	Tirz. 12.5	2 weeks	39	88	75	79	32	27	29
6	68	F	Sema. 2.4	10 days	33	86	74	72	29	25	24
7	46	F	Tirz. 5	2 weeks	58	88	76	75	30	26	26
8	39	M	Sema. 1.7	2 weeks	37	101	86	85	30	25	25
9	67	M	Sema. 1.7	2 weeks	54	88	80	79	27	25	24
10	63	M	Sema. 2.4	2 weeks	37	91	85	83	30	28	27
11	61	M	Tirz. 7.5	2–3 weeks	32	80	67	70	27	23	24
12	50	M	Tirz. 15	2 weeks	11	113	95	89	34	29	27
13	77	M	Tirz. 7.5	5–6 weeks	54	110	83	82	37	28	27
14	77	F	Tirz. 5	2 weeks	24	80	62	55	32	25	22
15	71	M	Sema. 2	10 days	40	87	70	74	32	25	27

Case	Pre A1c	Plat A1c	Maint A1c	Pre Tg	Plat Tg	Maint Tg	Pre HDL	Plat HDL	Maint HDL	Pre BP	Plat BP	Maint BP
1	5.2	5.3	5.1	47	35	46	94	98	96	125/82	99/66	124/62
2	6.4	5.7	5.6	100	139	118	61	58	58	105/66	103/66	97/60
3	5	4.5	5	120	62	71	40	41	53	112/71	104/69	124/76
4	7.2	5.8	5.7	95	78	72	47	36	46	124/69	112/67	119/66
5	5.2	5	5.1	110	65	59	42	54	58	114/82	120/83	153/81
6	5.1	4.7	4.9	96	92	103	53	48	53	121/78	100/68	124/86
7	5.3	5.1	5	34	85	65	75	69	67	124/73	95/60	95/60
8	6.1	5.4	4.8	284	101	85	37	42	58	105/64	115/80	118/79
9	5.6	5.3	4.9	74	62	70	74	68	69	138/75	127/74	108/69
10	5.2	4.9	4.7	205	61	51	55	53	58	154/89	122/85	127/82
11	5.3	5	4.8	132	73	74	42	48	55	103/59	90/53	100/65
12	5.7	4.8	5.5	98	58	53	48	42	55	147/99	127/83	111/78
13	8	5.2	5.1	110	103	84	40	34	47	156/76	124/67	121/69
14	5.5	—	5	68	—	57	50	—	52	125/72	132/86	93/62
15	5.7	5.5	5.5	98	67	51	44	40	38	113/67	130/69	126/70

Case	Age	Sex	GLP1 dose	Frequency	Weeks maint	Pre Wt	Plat Wt	Maint Wt	Pre BMI	Plat BMI	Maint BMI
16	50	M	Tirz. 15	10 days	18	92	84	81	28	26	25
17	53	F	Tirz. 7.5	3 weeks	19	79	63	61	27	22	21
18	85	M	Tirz. 15	2 weeks	38	90	83	84	29	27	27
19	56	F	Tirz. 2.5	2 weeks	27	65	59	57	25	23	22
20	35	M	Tirz.7.5	2 weeks	26	93	74	72	32	25	25
21	47	F	Tirz.5	12–14 days	17	71	65	62	26	24	23
22	55	F	Tirz. 7.5	3 weeks	16	74	59	58	31	25	24
23	59	M	Tirz. 5	3 weeks	33	108	94	86	32	28	26
24	77	F	Tirz.15	2 weeks	31	78	62	59	28	22	21
25	69	M	Sema. 0.5	2 weeks	60	81	75	75	24	22	22
26	58	F	Tirz.15	2 weeks	25	89	65	61	29	21	20
27	74	M	Sema. 1	3 weeks	25	115	107	103	33	31	29
28	43	F	Tirz. 12.5	2 weeks	26	98	73	70	33	24	24
29	34	F	Tirz. 15	10 days	27	107	86	78	42	34	30
30	33	F	Sema. 1.7	3 weeks	14	67	53	53	27	21	21

Case	Pre A1c	Plat A1c	Maint A1c	Pre Tg	Plat Tg	Maint Tg	Pre HDL	Plat HDL	Maint HDL	Pre BP	Plat BP	Maint BP
16	5	—	5	60	—	46	42	—	52	114/65	98/61	124/65
17	5.1	—	—	108	—	115	54	—	68	132/80	100/66	103/68
18	5.6	5.7	5.1	92	52	55	46	45	50	99/65	107/70	119/68
19	6.5	5.7	5.7	262	283	109	47	36	43	104/69	110/71	95/66
20	4.4	4.2	4.1	160	69	98	34	36	35	119/81	130/89	113/76
21	—	4.5	4.9	—	48	73	—	56	63	—	91/55	109/65
22	5.7	—	5.4	118	—	76	77	—	74	122/78	—	108/77
23	5.4	4.7	4.7	107	69	61	34	36	39	150/79	124/84	108/70
24	5.9	5.1	5.2	169	96	76	50	56	58	145/76	111/55	111/59
25	5.9	5.3	5.3	167	126	76	59	60	71	128/73	135/71	132/69
26	5.1	4.6	4.9	65	51	45	53	58	68	106/73	110/78	130/85
27	5.2	4.9	5.1	89	70	93	58	44	48	144/73	110/72	121/68
28	5.3	4.7	4.9	147	69	96	39	36	40	102/60	90/63	116/66
29	5.9	5.5	5.3	227	94	115	41	43	48	117/82	109/77	108/76
30	5.2	—	4.9	67	—	50	64	—	59	107/67	95/69	99/69

*Note*: Case data of patients on reduced‐frequency tirzepatide (Tirz.) and semaglutide (Sema.). *Green* BMI < 25 kg/m^2^, HbA1c < 5.7%, triglycerides < 150 mg/dL, HDL > 50 mg/dL in women or > 40 mg/dL in men, normotensive (< 120/80 mmHg). *Yellow* 25 ≤ BMI < 30 kg/m^2^, 5.7% ≤ HbA1c < 6.5%, 150 ≤ triglycerides < 200 mg/dL, elevated blood pressure (120–129/< 80 mmHg). *Red* BMI ≥ 30 kg/m^2^, HbA1c ≥ 6.5%, triglycerides ≥ 200 mg/dL, HDL ≤ 50 mg/dL in women or ≤ 40 mg/dL in men, hypertension (≥ 130/80 mmHg).

The primary endpoint of weight was observed for all 30 patients at all time points. However, given the observational nature of our study, secondary endpoints, which required patients to complete lab studies or body composition scanning, saw losses to follow‐up such that metabolic lab studies were only completely obtained for 24 patients and body composition analysis at the maintenance time point for 20 patients.

### 3.2 Weight

**FIGURE 2:**
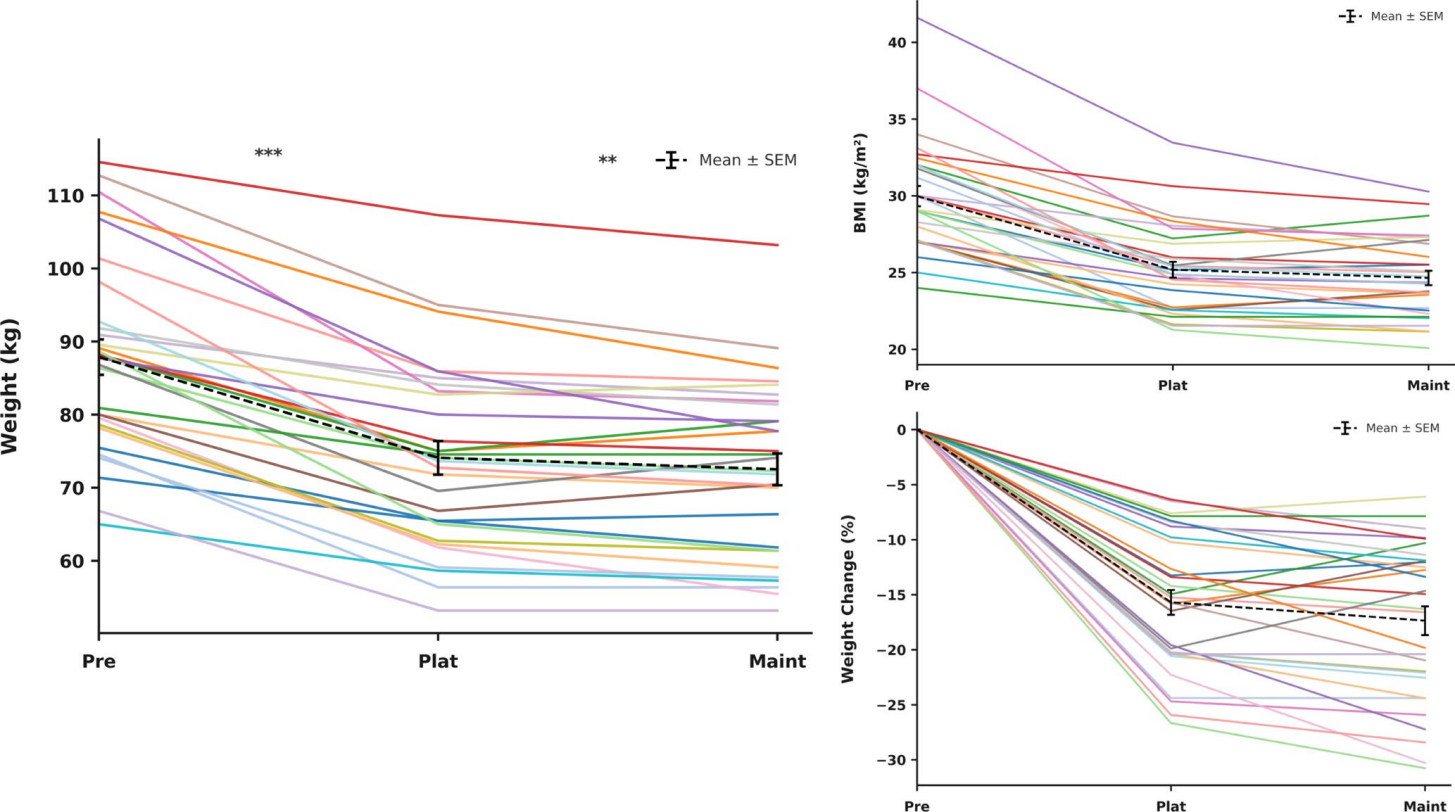
Weight trends for individual patients with patient average plotted (black), represented by absolute weight (kg, left) and BMI (kg/m^2^) and percentage weight loss from baseline/pre‐GLP1 treatment (right). Paired *t,*  < 0.01**, < 0.001***, *n* = 30. [Color figure can be viewed at wileyonlinelibrary.com]

During the weekly dosing regimen, GLP1 medications significantly reduced body weight in the 30 patients from a mean of 87.9 ± 2.4 kg at pre‐GLP1 treatment to 74.1 ± 2.4 kg (*p* < 0.001) at the plateau time point, which was a percent change of −17.2% ± 1.3% and a change in BMI from 30.0 ± 0.7 to 25.2 ± 0.5 kg/m^2^ [Correction added on 13 March 2026, after first online publication: In the preceding sentence, “26 patients” has been corrected to “30 patients.”]. While on maintenance dosing, patients exhibited a small further weight loss to 72.4 ± 2.2 kg (*p* < 0.01) and BMI 24.6 ± 0.5 kg/m^2^ for a difference from plateau of an additional −2.3% ± 0.7% (Figure 2).

### 3.3 Body Composition

**FIGURE 3:**
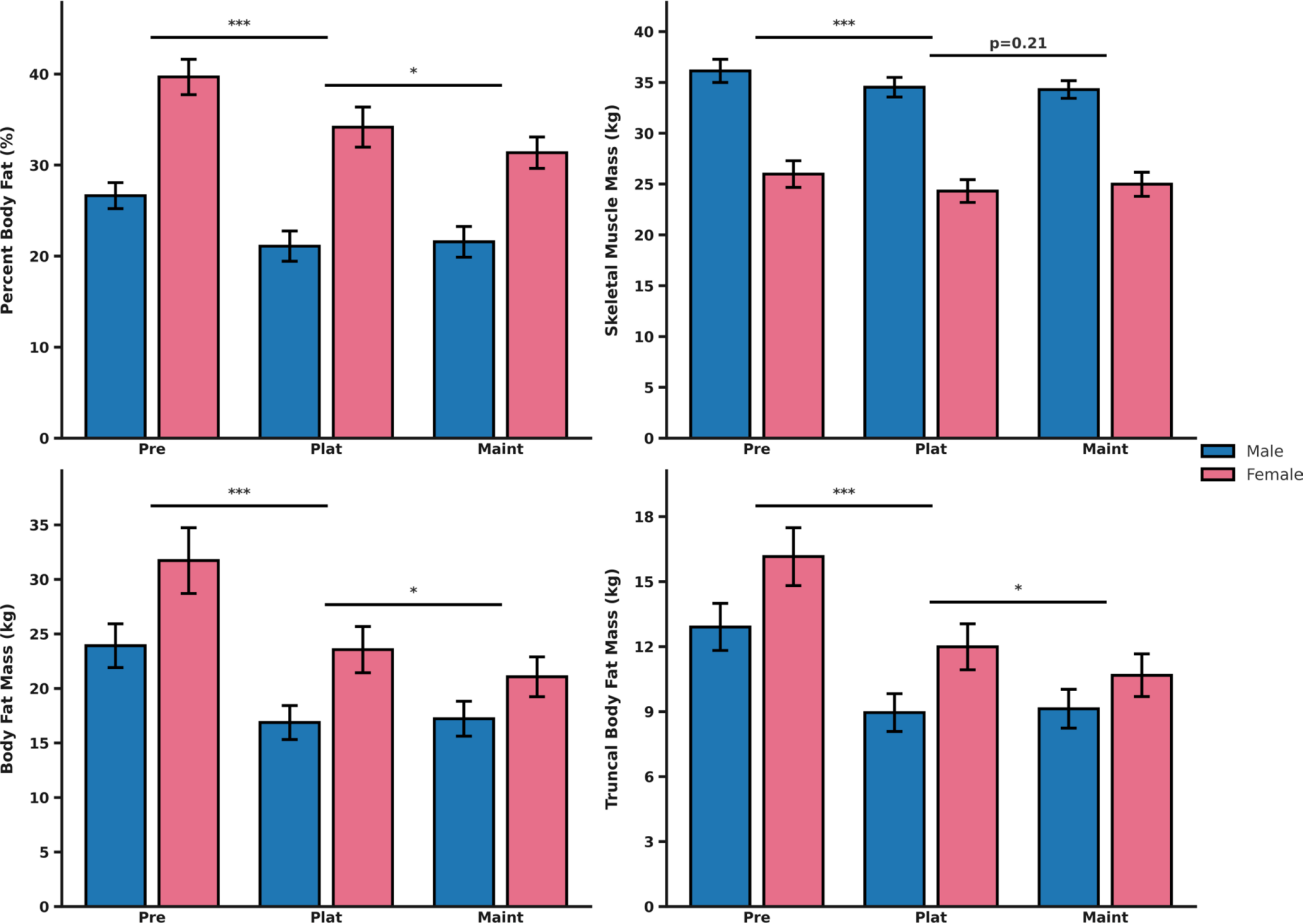
Body composition analysis for percent body fat as well as total body fat, truncal body fat, and skeletal muscle mass (kg) derived from bioelectric impedance separated by sex. Paired *t, p* < 0.05*, < 0.001***. Pre *n* = 23, Plat *n* = 24, Maint *n* = 20. [Color figure can be viewed at wileyonlinelibrary.com]

During the weekly dosing regimen, GLP1 medications significantly reduced percent body fat, body fat mass, truncal body fat mass, and skeletal muscle mass (*p* < 0.001, Figure 3). The average percent body fat decreased from 26.6% ± 1.4% to 21.1% ± 1.7% in men and from 39.7% ± 1.9% to 34.2% ± 2.2% in women. These changes were accompanied by a change in absolute body fat mass from 24.0 ± 2.0 kg to 16.9 ± 1.6 kg in men and from 31.8 ± 3.0 kg to 23.6 ± 2.1 kg in women. Skeletal muscle mass loss was statistically significant but proportionally smaller during weekly treatment, with a change from 36.2 ± 1.1 kg to 34.6 ± 1.0 kg in men and from 26.0 ± 1.3 kg to 24.4 ± 1.1 kg in women. This represents a ratio of weight loss of 5.3:1 fat to muscle in men and women. During the maintenance phase of treatment, average percent body fat, body fat mass, and truncal body fat mass showed continued small but significant declines (*p* < 0.05) while skeletal muscle mass trended toward increase (*p* = 0.21).

### 3.4 Metabolic Syndrome

**FIGURE 4:**
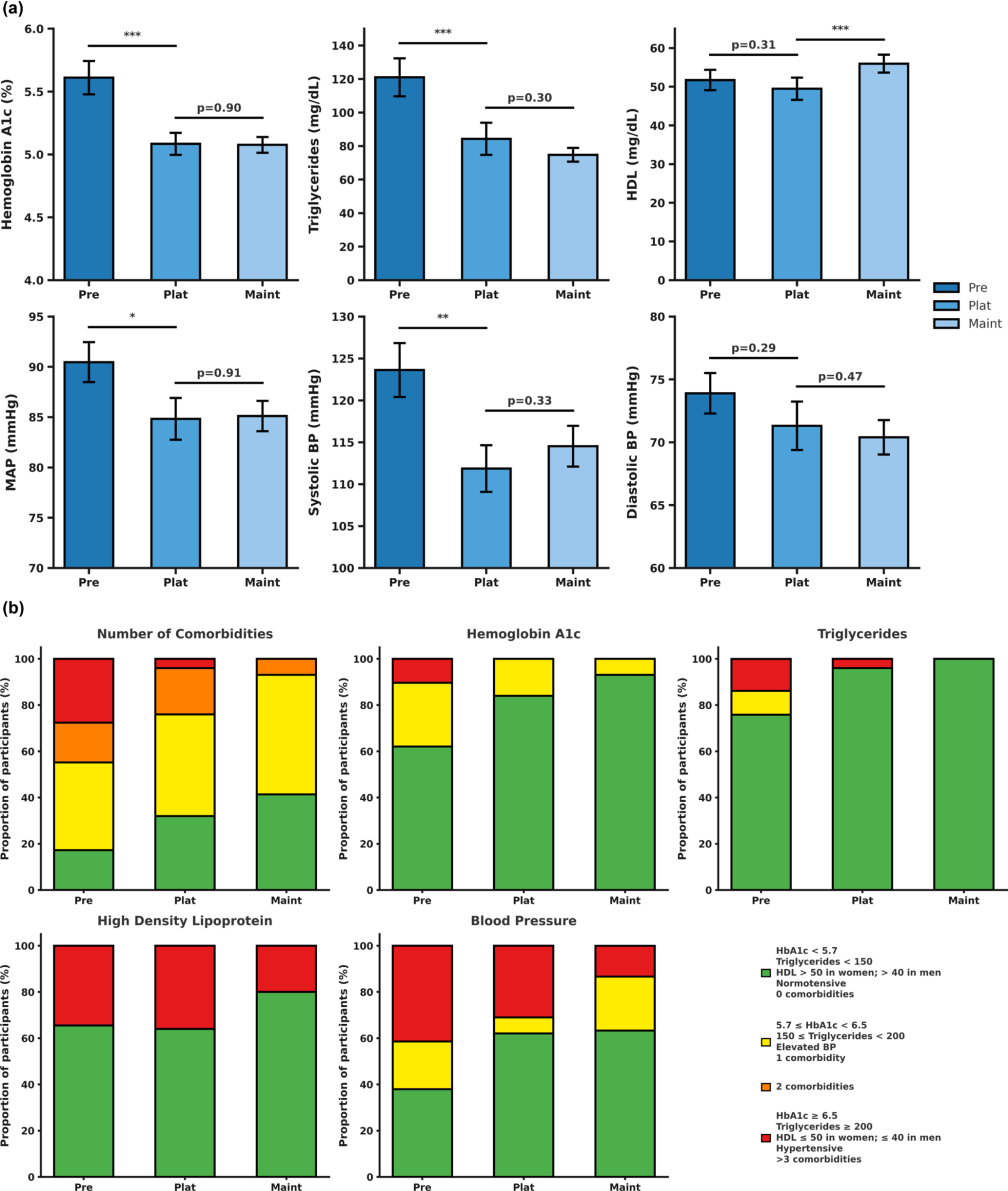
Metabolic syndrome parameters represented as (a) average of continuous measures with paired *t, p* < 0.05*, < 0.01**, < 0.001***, *n* = 25–29 or (b) categorical prevalence at each time point as percentage of patients. *Green* BMI < 25 kg/m^2^, HbA1c < 5.7%, triglycerides < 150 mg/dL, HDL > 50 mg/dL in women or > 40 mg/dL in men, normotensive (< 120/80 mmHg). *Yellow* 25 ≤ BMI < 30 kg/m^2^, 5.7% ≤ HbA1c < 6.5%, 150 ≤ triglycerides < 200 mg/dL, elevated blood pressure (120–129/< 80 mmHg). *Red* BMI ≥ 30 kg/m^2^, HbA1c ≥ 6.5%, triglycerides ≥ 200 mg/dL, HDL ≤ 50 mg/dL in women or ≤ 40 mg/dL in men, hypertension (≥ 130/80 mmHg). [Color figure can be viewed at wileyonlinelibrary.com]

During weekly dosing, improvements in components of metabolic syndrome—prediabetes, triglycerides, HDL, and blood pressure—were observed, and these improvements were retained while on reduced‐frequency maintenance dosing (Figure 4a). Mean HbA1c prior to GLP1 initiation was 5.6% ± 0.13%, which significantly improved to 5.1% ± 0.1% following weekly dosing (*p* < 0.001) and was unchanged at 5.1% ± 0.1% following reduced‐frequency dosing (*p* = 0.89). Mean triglycerides began at 121.0 ± 11.3 mg/dL, significantly improved to 84.3 ± 9.6 mg/dL (*p* < 0.001) following weekly dosing, and trended toward continued reduction while on reduced‐frequency dosing (74.8 ± 4.1 mg/dL, *p* = 0.29). Similarly, there was a reduction in mean arterial blood pressure from 90.5 ± 2.0 mmHg before to 84.8 ± 2.1 mmHg after weekly dosing (*p* < 0.05), which remained unchanged on maintenance dosing (85.1 ± 1.5 mmHg, *p* = 0.91). HDL levels were unchanged while on standard dosing (from 51.7 ± 2.6 to 49.5 ± 2.9 mg/dL, *p* = 0.31) but significantly improved to 56.0 ± 2.3 mg/dL with reduced‐frequency dosing (*p* < 0.001).

Overall comorbidity burden assessed by clinical diagnostic cut point prevalence was also improved over time while on standard dosing and reduced‐frequency dosing (Figure 4b). Prior to GLP1 initiation, the prevalence of individual weight‐related comorbidities was 37.9% for HbA1c ≥ 5.7%, 24.1% for triglycerides ≥ 150 mg/dL, 33.3% for HDL ≤ 40 for men and ≤ 50 mg/dL for women, and 62.1% for systolic blood pressure ≥ 120 or diastolic blood pressure ≥ 80 mmHg. In total, 82.8% of patients had at least one of these comorbidities prior to treatment. Following initiation of weekly GLP1, prevalence of individual comorbidities decreased, and the percentage of patients with any comorbidity decreased to 68.0%. During the maintenance phase of treatment, the prevalence of patients with any metabolic syndrome comorbidity declined further to 58.6%.

## 4 Discussion

GLP1 therapies have transformed obesity treatment, but barriers such as cost, drug supply, insurance coverage, and concerns about long‐term use continue to limit widespread adoption. To date, no published studies have examined patient outcomes using reduced‐frequency or maintenance dosing regimens following initial weight loss, and no standard of care exists for individuals seeking to de‐escalate therapy.

In this retrospective case series, we report outcomes from patients who transitioned to reduced‐frequency, typically nonmaximal GLP1 dosing after their weight plateaued and metabolic syndrome comorbidities improved on standard weekly therapy. We hypothesized that these improvements would be maintained following the transition. Our findings support this: patients maintained improvements in weight and had preserved gains in secondary outcomes such as body composition and metabolic markers.

This study differs from prior GLP1 de‐escalation trials in two key ways: (1) patient selection was based on clinical improvement rather than a fixed treatment duration, and (2) patients continued their current effective dose at reduced frequency rather than abruptly discontinuing therapy.

Regarding clinical readiness for de‐escalation, obesity is well established as a chronic disease with recognized compensatory mechanisms for appetite regulation and energy expenditure [25] that drive weight regain [26] and persist for years [27] after successful weight loss [28]. Data from the STEP 1 extension [11] and STEP 4 [12] trials demonstrate significant weight regain following GLP1 discontinuation after fixed treatment durations (68 and 20 weeks, respectively), despite substantial weight loss (17.3% and 10.6%), and BMI levels that remained above 30 at the time of cessation. Notably, weight did not fully return to baseline in the STEP 1 extension, suggesting possible benefit from longer therapy duration. In our study, by introducing reduced‐frequency dosing only after patients had experienced significant weight loss plateau (which coincided with patients attaining an average BMI of 25 and improved metabolic comorbidities), we postulate that participants were less affected by the physiological drivers of regain. Future studies measuring incretin levels and energy expenditure at the time of de‐escalation could further validate this theory.

Our de‐escalation approach—reducing frequency rather than fully discontinuing therapy—also aligns with behavioral literature on weight maintenance. Behavioral strategies commonly observed in individuals who successfully maintain weight loss—such as regular physical activity [29]—are associated with relatively small but meaningful changes in GLP1 and other incretin hormones. These changes are modest compared to those induced by potent interventions that drive initial weight loss. For example, monitor‐verified moderate‐to‐vigorous exercise increased GLP1 levels by 37% [22], while exclusive consumption of ultraprocessed foods reduced GLP1 by 34% [21]. These shifts, although notable, are still modest compared to pharmacologic GLP1 levels or the 10‐fold increases seen after bariatric surgery [30, 31]. Our findings suggest that while large increases in GLP1 levels may be necessary to induce weight loss, more modest elevations may be sufficient to maintain it.

This case series has several limitations. Selection bias may be present, as patients opting to de‐escalate therapy may also be more motivated to adopt behavioral strategies. Participants were not blinded and were aware of their reduced‐frequency schedule, which could further influence outcomes. While weight data were available for all patients, follow‐up for secondary endpoints (e.g., labs, body composition) had greater attrition due to higher patient burden. The average BMI in our cohort was 30—near the national average [32]—but only two participants had class 2 or 3 obesity, limiting generalizability to these populations. Generalizability to non‐White populations was also limited, as 26 of 30 patients were White. Finally, this observational case series includes only outcomes of patients on reduced‐frequency dosing; an important future direction for this work would compare these outcomes in a controlled trial either to individuals in a placebo group or to those who were offered but never started a reduced‐frequency schedule or those who started but ultimately returned to weekly therapy due to weight regain (4 of our 34 initial patients).

Although continued maintenance of health outcomes despite reducing therapy is a high standard for any medication, it remains the most common question we receive from patients initiating GLP1 treatment: “Will I have to take this forever?” The lack of evidence‐based guidance contributes to hesitancy. Notably, similar questions are rarely asked about other long‐term medications such as antihypertensives or hormone replacements. Few patients expect to take blood pressure medication only a few days per week or to discontinue thyroid or testosterone therapy once stabilized. GLP1 therapy, however, is often held to a higher standard—perhaps due to its relative novelty in public consciousness or persistent weight stigma that questions whether obesity merits chronic medical treatment. Remarkably, our findings suggest that GLP1 medications can meet this elevated expectation. Many patients in our study maintained outcomes on reduced‐frequency regimens, providing early evidence that structured de‐escalation may be both feasible and effective—unlike most chronic therapies, where such a goal is rarely pursued.

## 5 Conclusion

Our findings demonstrate that many patients who initially lose weight on standard weekly GLP1 therapy maintain weight, body composition, and metabolic parameters after transitioning to reduced‐frequency dosing. This study provides early proof of concept that structured de‐escalation may be a viable strategy to sustain benefits while reducing treatment burden. Larger randomized controlled trials are needed to confirm these findings and may help address concerns about indefinite therapy, lower health care costs, ease supply constraints, and broaden access to GLP1 medications to improve public health.

## Funding

This study was supported by a research award from the Scripps Clinic Medical Group to M.B.

## Conflicts of Interest

M.B. is a clinical trial site investigator for GLP1 agents for Eli Lilly and Company and Novo Nordisk. The other authors declare no conflicts of interest.

## Data Availability

The data that support the findings of this study are available on request from the corresponding author. The data are not publicly available due to privacy or ethical restrictions.
